# The role of inflammation in the relationship of self-rated health with mortality and implications for public health: Data from the English Longitudinal Study of Aging (ELSA)

**DOI:** 10.1016/j.bbih.2020.100139

**Published:** 2020-09-08

**Authors:** Sunjai Gupta, Yin Xu, Scott Montgomery

**Affiliations:** a Freelance Researcher, London, United Kingdom; b Clinical Epidemiology and Biostatistics, School of Medical Sciences, Örebro University, Sweden; c Clinical Epidemiology Division, Karolinska Institutet, Stockholm, Sweden; d Department of Epidemiology and Public Health, University College London, UK

**Keywords:** Inflammation, Self-reported health, Mortality, ELSA, Older adults, SRH, self-reported health, CRP, C-reactive protein, ELSA, English Longitudinal Study of Ageing, ASCVD, atherosclerotic cardiovascular disease, ESR, Erythrocyte Sedimentation Rate, BMI, body mass index

## Abstract

Self-rated health (SRH) predicts mortality after adjustment for potential confounders, including measures of health status. Prodromal disease might lead to worsened SRH and higher mortality. But no study of SRH and mortality has focussed on inflammation. The objective of this study is to investigate the influence of inflammation upon the association between SRH and mortality in a British cohort. The English Longitudinal Study of Ageing (ELSA) involves interviewing participants aged over 50 every two years. We analysed data for 3405 men and 4139 women. Mortality for consenting members was detected by linkage with UK National Health Care registry up to March 2012. Demographic, clinical, and health behaviours at wave 2 were treated as confounders, as well as inflammation-related disease and C-reactive protein (CRP). A five-step hierarchical multivariable logistic regression was estimated. An association was observed between SRH and mortality after adjusting for all variables. In men, compared to those with excellent health, CRP only, and CRP and inflammation-related disease combined, could explain 7.03% and 24.35% of increased risk of dying associated with poor health, respectively. For women, the corresponding figures were 8.95% and 24.28%, respectively. Inflammation is associated with increased risk of death, and may help to explain approximately a quarter of the association between SRH and mortality. Individuals with relatively poor SRH may be aware of underlying inflammation that increases the risk of illness and death, and this may lead to increased use of services, for example. Identifying the cause and treating inflammation in those without a diagnosis may help to increase survival and life quality among those who perceive their health to be relatively poor.

## Highlights

• Poor self-rated health (SRH) is associated with increased risk of death.
• In a prospective study, inflammation explained 1/4 of the association.
• Identifying inflammation early may improve survival and life quality.
• SRH can assist management, inform policy and guide research.

## 1 Introduction

The association between self-rated health (SRH) and mortality has been the subject of considerable interest and debate. Furthermore, the majority of studies in this area have found that this relationship remained even after explanatory variables, including measures of health, were controlled for (Idler and Benyamini, 1997).

SRH may reflect underlying disease that is in a “prodromal” state (Idler and Benyamini, 1997). This might manifest in biological measures and, after allowing for a wide range of such objective indices, it tends only to be those with the worst SRH who still have increased mortality (Jylha, 2009). If SRH does, at least in part, reflect underlying biological malaise, then it should correlate with biological measures. For example, Bello et al. developed a “Health Status metric” which included biomarkers such as C-reactive protein (CRP) (Bello et al., 2015). It predicted all-cause mortality, and was associated with concurrent SRH and with various self-reported health conditions (e.g. diabetes).

Haring et al. measured SRH and a range of self-reported conditions (again including diabetes, for example), and biomarkers amongst which were high sensitive CRP (Haring et al., 2011). Relatively poor SRH was related to higher risk of death and this was not substantially altered by controlling confounders including biomarkers.

Barger et al. calculated 10 year “atherosclerotic cardiovascular disease (ASCVD) risk” which included, for instance, high blood pressure and a history of diabetes (Barger et al., 2016). SRH predicted all cause mortality even after allowing for ASCVD risk and non-conventional biomarkers including CRP. They suggest that SRH measures health in a way that is reflected in part by known biological measures but which also extends further than these.

There is an association between systemic inflammation and mortality, even where the inflammation is measured in late adolescence among ostensibly healthy individuals (Kantor et al., 2019). Furthermore, even modestly raised CRP levels can indicate undiagnosed subclinical diseases processes that are relevant to mortality. Some of the above studies included measures of immune function, and others have shown an association between SRH and measures of immunological status. For example, Warnoff et al. (2016) examined the relationship of Erythrocyte Sedimentation Rate (ESR) to SRH after allowing for various confounders and inflammation-related diseases including cancer, diabetes mellitus and asthma. Increased ESR was associated with relatively poor SRH, but the authors acknowledge that the direction of causation between ESR and SRH could not be determined given the cross-sectional nature of their study.

Christian et al. have also recommended using prospective studies which include measures of SRH, inflammation and mortality (Christian et al., 2011). Such studies could test whether dysfunction of the immune system might help explain the association between SRH and mortality (Uchino et al., 2019), but, to our knowledge, no study to date has done this. We decided to examine this question using data from the English Longitudinal Study of Ageing (ELSA) which included a measure of immune status (CRP) and also information about a wide range of inflammation-related diseases.

## 2 Materials and methods

### 2.1 Participants

The English Longitudinal Study of Ageing (ELSA) is based on data collected from participants aged over 50, who originally took part in the Health Survey for England which is an annual national survey and is used to monitor the health of the population to facilitate the development of policy (Gupta, 1994). ELSA was approved by the appropriate ethics committee and conformed to the principles embodied in the Declaration of Helsinki.

Starting in 2002, ELSA involves re-interviewing participants approximately every two years. It includes a broad range of measures including those of physical and mental health and wellbeing. ELSA is broadly representative of the population of older people in England. Comparisons of the socio-demographic characteristics of the participants against results from the national census indicate that the sample was representative (Steptoe et al., 2013). It is used to inform policy on ageing, for example relating to health and social care. https://www.elsa-project.ac.uk/.

Participants who completed interviews and were eligible for a nurse visit at wave 2 were included (N ​= ​7666). 82% of those who took part in wave 1 were successfully re-interviewed in wave 2. And 87% of those completed the interviews joined the nurse visit at wave 2. However, 122 participants did not give permission to check their mortality. As a result, 7544 (3405 men and 4139 women) were included in the analysis.

### 2.2 Measures

At wave 2, participants reported their current health on a 5-point scale (1 ​= ​excellent, 2 ​= ​very good, 3 ​= ​good, 4 ​= ​fair, 5 ​= ​poor). Mortality for consenting study members was detected by linkage with the UK National Health Service mortality registry up to March 2012. Several demographic, clinical, and health behaviour measures at wave 2 were treated as potential confounders (See Table 1). These included age, ethnicity (white or non-white), socio-economic classification, total wealth quintiles, marital status (married, cohabiting, single, widowed, divorced, and separated), body mass index (BMI), 7-CVD related diseases (arrhythmia, myocardial infarction, congestive heart failure, angina, heart murmur, diabetes or high blood sugar, stroke), other chronic diseases (asthma, arthritis, and chronic lung disease), cancer, hypertension, smoking, alcohol consumption, positive social support, physical activity, and C-reactive protein (CRP). Age (years) was divided into three categories (50–60, 61–70, and≥71) because those older than 90 were collapsed into one category in the original file. Socioeconomic position was measured using the National Statistics Socioeconomic Classification (managerial and professional occupations, intermediate occupations, and routine and manual occupations). Total.

**Table 1:** Characteristics of participants stratified by sex and mortality.

	Men		Women	
	Alive	Dead	Alive	Dead
Self-report health N (%)				
Excellent	412 (14.93)	30 (4.69)	480 (13.51)	25 (4.27)
Very good	827 (29.96)	99 (15.47)	1107 (31.17)	102 (17.41)
Good	904 (32.75)	213 (33.27)	1118 (31.47)	186 (31.73)
Fair	476 (17.25)	193 (30.16)	656 (18.47)	181 (30.89)
Poor	141 (5.11)	105 (16.41)	191 (5.38)	92 (15.70)
Ethnicity N (%)				
White	2698 (97.72)	635 (98.76)	3492 (98.31)	584 (99.83)
Non-white	63 (2.28)	8 (1.24)	60 (1.69)	1 (0.17)
Age (years) N (%)				
50-60	1105 (40.01)	67 (10.42)	1373 (38.64)	42 (7.17)
61-70	1011 (36.60)	125 (19.44)	1228 (34.56)	85 (14.51)
≥71	646 (23.39)	451 (70.14)	952 (26.79)	459 (78.32)
Marital status N (%)				
Married	2143 (77.59)	407 (63.29)	2182 (61.41)	207 (35.33)
Cohabiting	104 (3.77)	18 (2.80)	126 (3.55)	5 (0.85)
Single	136 (4.92)	30 (4.67)	128 (3.60)	33 (5.63)
Widowed	188 (6.81)	130 (20.22)	715 (20.12)	300 (51.20)
Divorced	152 (5.50)	45 (7.00)	336 (9.46)	37 (6.31)
Separated	39 (1.41)	13 (2.02)	66 (1.86)	4 (0.68)
Wealth quintile N (%)				
Lowest quintile	342 (12.56)	152 (23.64)	570 (16.31)	186 (31.86)
2nd quintile	496 (18.22)	143 (22.24)	685 (19.61)	133 (22.77)
3rd quintile	568 (20.86)	122 (18.97)	739 (21.15)	109 (18.66)
4th quintile	618 (22.70)	130 (20.22)	736 (21.06)	84 (14.38)
Top quintile	699 (25.66)	96 (14.93)	764 (21.87)	72 (12.33)
Socioeconomic position N (%)				
Managerial and professional	1111 (40.34)	208 (32.50)	918 (26.30)	110 (19.96)
Intermediate	544 (19.75)	140 (21.88)	1045 (29.94)	144 (26.13)
Routine and manual	1099 (39.91)	292 (45.62)	1527 (43.75)	297 (53.91)
BMI (kg/m^2^) N (%)				
Underweight	8 (0.30)	12 (2.14)	25 (0.73)	16 (3.28)
Normal weight	580 (21.85)	179 (31.96)	990 (29.03)	168 (34.43)
Overweight	1353 (50.96)	233 (41.61)	1329 (38.97)	170 (34.83)
Obese	714 (26.89)	136 (24.29)	1066 (31.26)	134 (27.46)
CVD N (%)				
No	1930 (69.88)	310 (48.21)	2685 (75.57)	305 (52.05)
Yes	832 (30.12)	333 (51.79)	868 (24.43)	281 (47.95)
Cancer N (%)				
No	2626 (95.08)	552 (85.85)	3281 (92.34)	498 (84.98)
Yes	136 (4.92)	91 (14.15)	272 (7.66)	88 (15.02)
Chronic disease N (%)				
No	1694 (61.33)	316 (49.14)	1693 (47.65)	201 (34.30)
Yes	1068 (38.67)	327 (50.86)	1860 (52.35)	385 (65.70)
Hypertension N (%)				
No	1632 (59.09)	317 (49.30)	2048 (57.64)	258 (44.03)
Yes	1130 (40.91)	326 (50.70)	1505 (42.36)	328 (55.97)
Smoking N (%)				
Current non-smoker	2362 (85.67)	528 (82.37)	3048 (86.00)	494 (84.30)
Current smoker	395 (14.33)	113 (17.63)	496 (14.00)	92 (15.70)
Alcohol consumption N (%)				
Almost every day	583 (23.13)	112 (21.54)	454 (13.96)	68 (14.85)
Five or six days a week	176 (6.98)	28 (5.38)	177 (5.44)	9 (1.97)
Three or four days a week	385 (15.27)	58 (11.15)	326 (10.02)	25 (5.46)
Once or twice a week	723 (28.67)	146 (28.09)	808 (24.84)	68 (14.85)
Once or twice a month	272 (10.79)	52 (10.00)	463 (14.23)	44 (9.61)
Once every couple of months	104 (4.13)	29 (5.58)	295 (9.07)	40 (8.73)
Once or twice a year	104 (4.13)	41 (7.88)	366 (11.25)	86 (18.78)
Not at all in the last 12 months	174 (6.90)	54 (10.38)	364 (11.19)	118 (25.75)
C-reactive protein (mg/l)				
Mean (SD)	0.61 (1.07)	1.06 (1.27)	0.71 (1.07)	1.09 (1.31)
Range	−1.61–5.35	−1.61–4.93	−1.61–4.91	−1.61–5.31
Skewness (Kurtosis)	0.30 (3.30)	0.14 (2.79)	0.12 (2.87)	0.30 (3.06)
Physical activity				
Mean (SD)	9.02 (2.30)	7.44 (2.78)	8.82 (2.20)	6.74 (2.53)
Positive social support				
Mean (SD)	23.28 (7.09)	21.69 (7.66)	23.84 (7.08)	21.09 (7.28)

Note. The range for C-reactive protein (log), physical activity, and positive social support is −1.61-5.35, 3–12, and 0–36, respectively. Normal distribution has a kurtosis of 3 and a skewness of 0. BMI ​= ​body mass index. CVD ​= ​cardiovascular disease.

Wealth quintiles were calculated using the sum of household financial, physical, and housing wealth minus mortgage, financial debts, and pension payments. BMI (kg/m^2^) was derived using measured height and weight, and was divided into four categories, underweight (<18.5), normal weight (18.5 to <25), overweight (25 to <30), and obese (≥30). Measures of 7-CVD related diseases, cancer, hypertension, and other chronic diseases were based on self-reported physician diagnoses. Smoking was classified into two groups (current smoker and current non-smoker) based on one item, whether the participants smoke cigarettes at all nowadays. Alcohol consumption was classified into eight categories (almost every day, five or six days a week, three or four days a week, once or twice a week, once or twice a month, once every couple of months, once or twice a year, and not at all in the last 12 months) based on one item, the frequency of drinking in the last 12 months. A measure of perceived positive social support was based on three items relating to perceived support from partner, children, relatives, and friends, respectively. Each item was measured on a 4-point scale (0 ​= ​not at all, 3 ​= ​a lot). The sum score of perceived positive support from partner, children, relatives, and friends was calculated and used in the analysis. Confirmatory factor analysis suggested that the four subscales measured one latent variable (x^2^ (50) ​= ​1396.00, *p* ​< ​.001, RMSEA ​= ​0.06, 90% confidence interval ​= ​[0.06, 0.07], CFI ​= ​0.94, TLI ​= ​0.93, SRMR ​= ​0.04). Participants reporting having no partner, children, relatives, or friends were given a value of 0. Physical activity was measured using three items (frequency of doing vigorous, moderate, and mild sports or activities) on a 4-point scale (1 ​= ​hardly ever or never, 4 ​= ​more than once a week). Exploratory factor analysis yielded one factor (eigenvalue ​= ​1.66) accounting for 55.37% of the variance in our sample. Levels of CRP (mg/l) were based on blood samples that were collected by study nurses.

### 2.3 Statistical analysis

The variables had 0.04–23.34% missing information within the analysis sample. These missing data were mainly due to missing blood samples (1435 without taking blood sample), and unlikely to be missing completely at random (e.g. physical activity, and cancer and chronic disease diagnoses predicted the missingness of BMI among women). This was handled using multiple imputation stratified by sex. All variables in the analysis were included for the imputation model. Twenty-four imputations were created to match the percentage of missing data (White et al., 2011). A chained equations algorithm model was used since a combination of categorical and continuous variables were with missing data. A predictive mean matching approach with 10 nearest-neighbour donors was used for continuous variables since this makes no distributional assumptions. After imputation, logistic regressions were performed in each imputed dataset separately and then the combined estimates using methods suggested by Rubin was calculated using estimates from 24 imputed datasets (White et al., 2011). Trace plots and other diagnostics provided no cause for concern regarding the imputed values. Sensitivity analysis was conducted to compare analyses based on complete-case analysis with analyses based on multiple imputation (see supplementary tables).

CRP was log-transformed before modelling since its distribution is highly skewed. For continuous covariates including CRP, positive social support, and physical activity, fractional polynomial analyses were performed to check the nature of the relation associated with mortality. Fractional polynomial suggested that positive social support, and physical activity were linearly associated with mortality, and CRP was non-linearly associated with mortality. Accordingly, the transformation suggested by fractional polynomial for CRP was used in the analyses, (CRP+1.61)^3^. Self-reported health was treated as categorical instead of continuous, confirmed via likelihood ratio test (*x*^*2*^ (3) ​= ​0.76, *p* ​= ​.86). There were also no multicollinearity problems based on the variance inflation factor (all <1.6).

In order to test whether self-reported health at wave 2 was associated with mortality between wave 2 and wave 5, and this association was explained by inflammation markers (CRP) measured at wave 2, a five-step hierarchical multivariable logistic regression was estimated. First, a univariate logistic regression was estimated with mortality regressed on self-reported health (Model 1). In the second step, demographic information, including age, ethnicity, marital status, socio-economic classification, and total wealth quintiles, was entered (Model 2). In the third step, risk factors, including BMI, smoking, alcohol consumption, positive social support, and physical activity, were entered (Model 3). In the fourth step, CRP was further entered (Model 4). In the final step, inflammation related diseases, including 7-CVD related diseases, other chronic diseases, cancer, and hypertension, were entered (Model 5).

Finally, in order to examine the role of CRP in the association between self-reported health and mortality among our sample without any inflammation-related diseases, we used *mimrgns* command to calculate the increased probability of death for CRP in Model 5 generated by the final step of the multivariable logistic regression. All inflammation-related diseases were set to zero and the remaining variables in Model 5 were set to the observed values. We were unable to run the five-step hierarchical multivariable logistic regression (described above) among our sample without any inflammation-related diseases due to small cell sizes (e.g., only 5 men without inflammation-related diseases reported poor health). All analyses were performed in Stata 16.0. Analyses were carried out separately for men and women.

## 3 Results

**Fig. 1:**
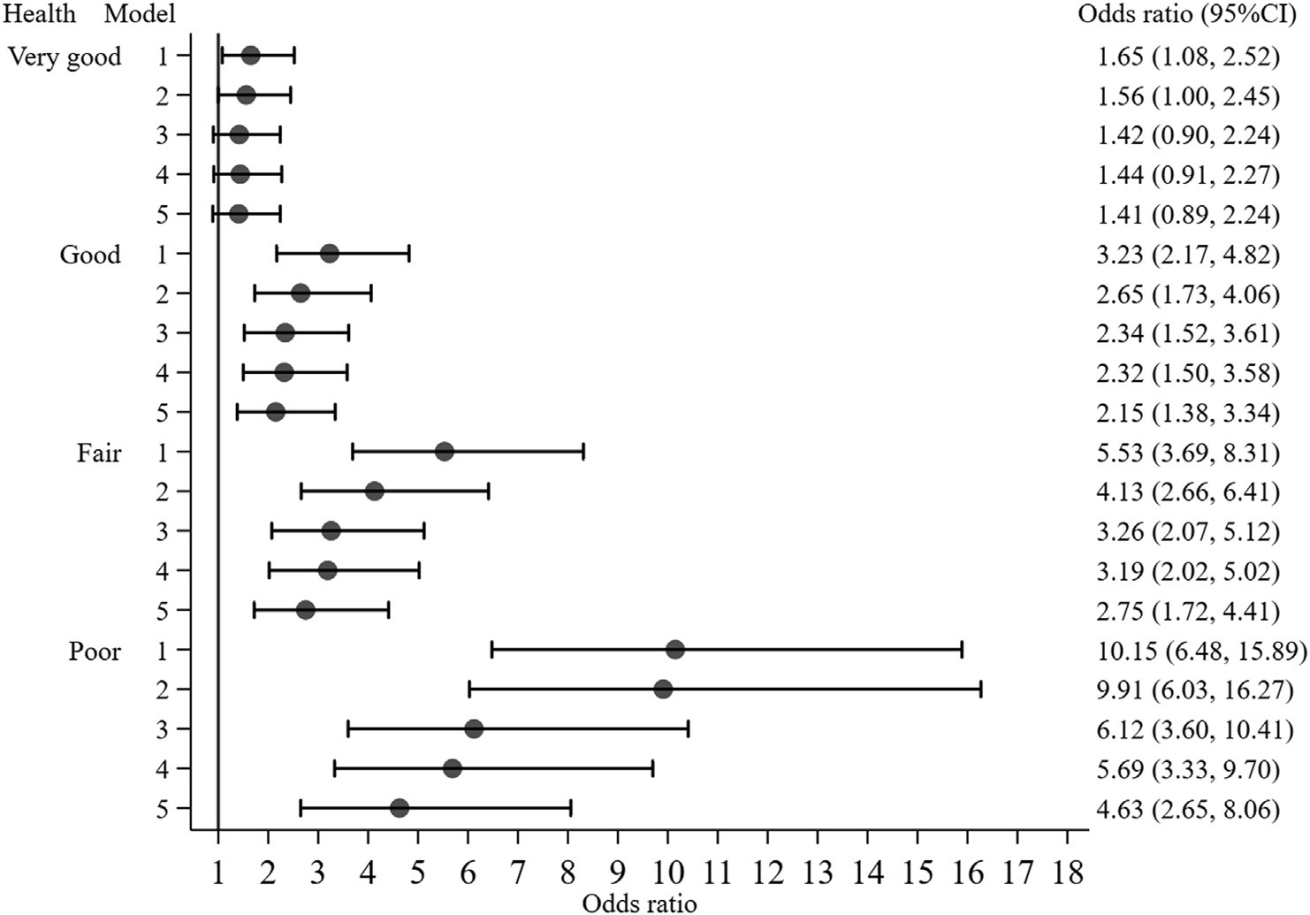
Odds ratio and 95% confidence interval for self-reported health from multiple imputation stratified by models among men. Note. Men with excellent health were the reference groups. Model 1 is the unadjusted model. Model 2 controlled for age, ethnicity, socio-economic classification, total wealth quintiles, and marital status. Model 3 is the same as Model 2 with body mass index, smoking, alcohol consumption, positive social support, and physical activity being further controlled for. Model 4 is the same as Model 3 with C-reactive protein being further controlled for. Model 5 is the same as Model 4 with 7-CVD related diseases, other chronic diseases, cancer, and hypertension being further controlled for.

The results show that there is a graded association between SRH and the risk of dying. Compared to men with excellent SRH, men with very good, good, fair, and poor health were at significantly greater risk of dying in the unadjusted model (Model 1), odds ratios range from 1.65 to 10.15. Compared to men with excellent SRH, the increased risk of dying associated with good, fair, and poor health became weaker but remained significant even after controlling for sociodemographic variables, risky behaviour, and measures of inflammation and inflammation related diseases (Model 5), with odds ratio ranging from 2.15 to 4.63 (Fig. 1). However, the significant increased risk associated with very good compared to excellent SRH among men disappeared after controlling for sociodemographic variables, risky behaviour, and measures of inflammation and inflammation related diseases (Fig. 1).

**Fig. 2:**
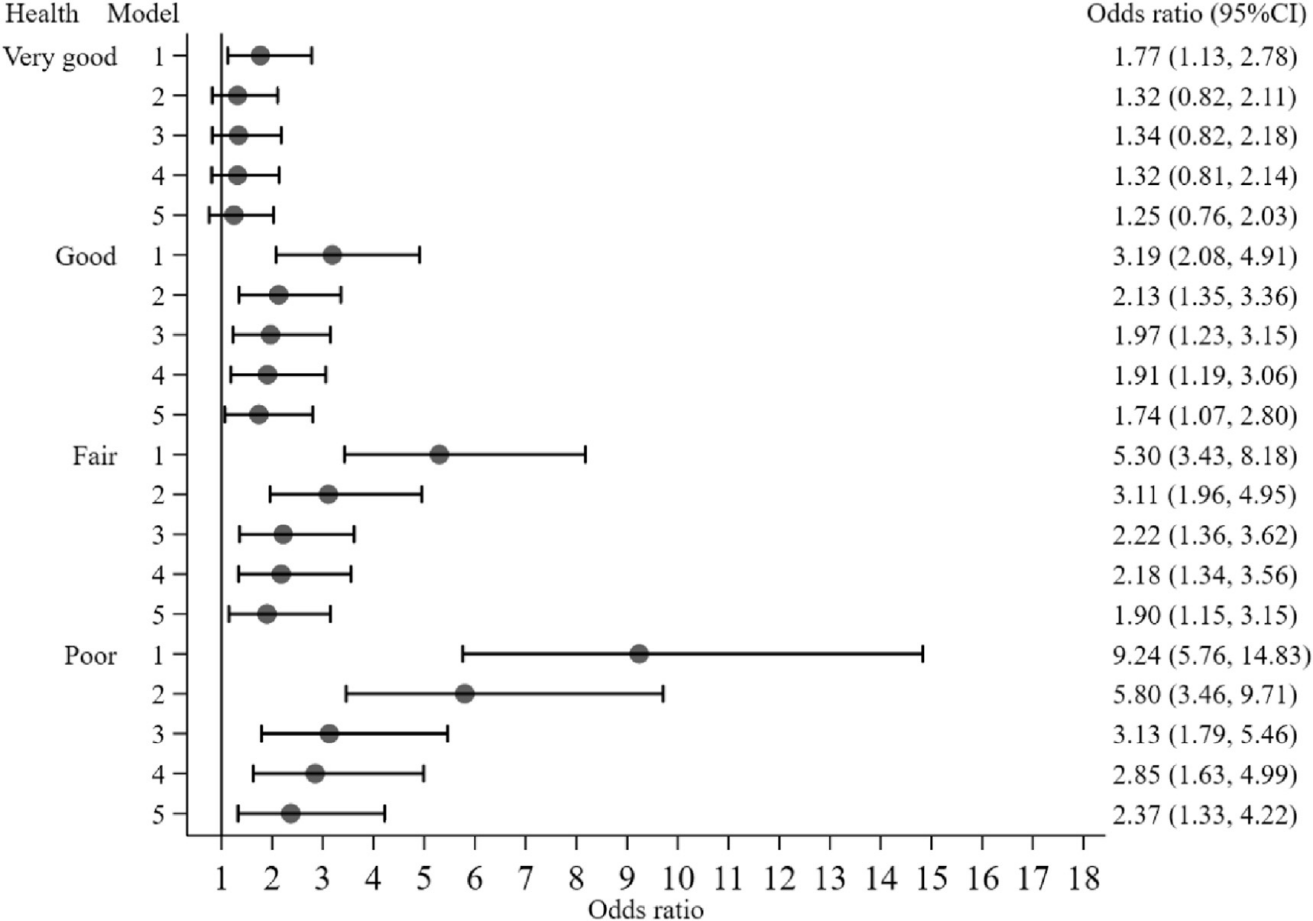
Odds ratio and 95% confidence interval for self-reported health from multiple imputation stratified by models among women. Note. Women with excellent health were the reference groups. Model 1 is the unadjusted model. Model 2 controlled for age, ethnicity, socio-economic classification, total wealth quintiles, and marital status. Model 3 is the same as Model 2 with body mass index, smoking, alcohol consumption, positive social support, and physical activity being further controlled for. Model 4 is the same as Model 3 with C-reactive protein being further controlled for. Model 5 is the same as Model 4 with 7-CVD related diseases, other chronic diseases, cancer, and hypertension being further controlled for.

Compared to women with excellent SRH, women with very good, good, fair, and poor health were at significantly greater risk of dying in the unadjusted model (Model 1), odds ratios range from 1.77 to 9.24. Compared to women with excellent SRH, the increased risk of dying associated with good, fair, and poor health became weaker but remained significant even after controlling for sociodemographic variables, risky behaviour, and measures of inflammation and inflammation related diseases (Model 5), with odds ratios ranging from 1.74 to 2.37 (Fig. 2). However, the significant increased risk associated with very good compared to excellent SRH among women disappeared after controlling for sociodemographic variables, risky behaviour, and measures of inflammation and inflammation related diseases (Fig. 2, Model 5).

CRP only (Model 3 v Model 4), and CRP and inflammation-related disease combined (Model 3 v Model 5) could explain 7.03% and 24.35% of the increased risk of dying associated with poor SRH compared to men with excellent SRH, respectively. For women, CRP only (Model 3 v Model 4), and CRP and inflammation-related disease combined (Model 3 v Model 5) could explain 8.95% and 24.28% of the increased risk of dying associated with poor SRH compared to women with excellent SRH, respectively.

CRP only (Model 3 v Model 4), and CRP and inflammation related disease combined (Model 3 v Model 5) could explain −1.41% and 0.70% of the increased risk of dying associated with very good SRH compared to men with excellent SRH, respectively. For women, CRP only (Model 3 v Model 4), and CRP and inflammation related disease combined (Model 3 v Model 5) could explain 1.49% and 6.72% of the increased risk of dying associated with very good SRH compared to women with excellent SRH, respectively.

One unit increase in CRP (log-transformed) was associated with 0.08% (95%CI ​= ​0.04%–0.12%) increase in mortality among men without any inflammation-related diseases. Similarly, one unit increase in CRP (log-transformed) was associated with 0.06% (95%CI ​= ​0.02%–0.09%) increase in mortality among women without any inflammation-related diseases.

## 4 Discussion

To our knowledge, this is the first study to measure the association between SRH and risk of death and also to specifically focus on a measure of systemic inflammation (CRP) and a range of inflammation-related diseases. Even after controlling for a range of measures including those of sociodemographic factors, risk behaviour and inflammation and inflammation-related diseases, there is a graded relationship between SRH and mortality risk. The latter remains statistically significantly elevated compared to those reporting excellent health, with the exception of those reporting very good health, in both men and women.

Furthermore, after allowing for a number of potential confounders, controlling for inflammation-related disease and CRP reduced by approximately 24% the elevated risk of death of those reporting poor health compared to those reporting excellent health in both sexes. The association between poor SRH and mortality appears, therefore, to be partially explained by diagnosed inflammation and inflammation-related diseases. Our analysis assumes that inflammation influences both SRH and mortality risk. This is supported by the idea that individuals may be aware of bodily processes (such as those related to inflammation; Andreasson et al., 2019; Kaplan and Camacho, 1983; Leshem-Rubinow et al., 2015; Stenback, 1964) which may increase the risk of poor health outcomes (Warnoff et al., 2016).

Moreover, such awareness may also influence behaviour. For example, in a study from Italy relatively poor SRH had a stronger association with hospitalisation than either age or the presence of health conditions, and of all the variables studied SRH showed the strongest relationship with the decision to ask for an examination by a medical specialist (Cislaghi and Cislaghi, 2019). Similarly, a study of asthmatics found that those who rated their health more poorly were more likely to have themselves vaccinated against seasonal influenza (Santos-Sancho et al., 2013). However, there are a number of studies which found that even after controlling for “health practices” the relationship of SRH and mortality remained (Idler and Benyamini, 1997). Moreover, in the present study, even after controlling for all confounders, the mortality risk remained significantly elevated in almost all groups compared to those reporting excellent health, so it is likely that mechanisms other than inflammation are involved.

### 4.1 Implications of SRH for health and public health

Our findings add to the body of evidence that SRH is related to inflammation and supports the idea that this may help to explain why SRH predicts mortality. In turn, therefore, they also strengthen the idea that SRH may be a useful tool for use at both an individual and at a population level. For example, Barger et al. maintain that the advantage of SRH is that no single set of known biomarkers can adequately assess health and that poor SRH could be used to guide further management (Barger et al., 2016). Similarly, Cohen et al. have argued that it could be used by clinicians alongside more conventional markers of such risk, especially since it is a harmless and cost-effective way of assessing disease predisposition particularly that linked to immune function (Cohen et al., 2015). Cho and Irwin (2015) also suggest that research should investigate the possibility that there may be interventions that could influence immune processes and thus reduce the risk of illness and death in sub-groups with relatively poor SRH. Uchino et al. show that, not only is relatively poor SRH associated with increased levels of C-reactive protein, but also that a key mediator of this relationship was sleep quality (though not depression) which, they argue, is potentially amenable to intervention (Uchino et al., 2019).

From a population perspective, one of the authors of the present paper (SG) has worked with others to use SRH in the development of a measure of “healthy life expectancy” which has the benefit of measuring health in a “positive” as well as in a negative sense (i.e. the presence or absence of a limiting longstanding illness, for example). Such measures have the potential for use in monitoring policy and assessing future demand for health and social services (Kelly et al., 2000).

### 4.2 Potential limitations

We could have used other measures of inflammation, e.g. ESR, but were constrained by the variables in the ELSA dataset. CRP levels may have reflected processes and severity associated with both diagnosed and undiagnosed/subclinical diseases. However, our analyses show that CRP was significantly associated with increased risk of mortality in both men and women without any inflammation-related diseases. We have not necessarily included all diseases which are thought to have an immunological component, or which might have such a component (Gupta, 1993; Khandaker et al., 2015). There may also have been under-reporting of diagnosed conditions. Since both SRH and inflammation were assessed only once at baseline, our current analysis also cannot exclude the possibility that SRH influences future inflammation which in turn then influences mortality (Idler and Benyamini, 1997). This is a potential avenue for further research. All of these factors could have led to an under-estimate of the role of inflammation caused by diagnosed diseases. On the other hand, there is evidence that SRH is not always independently and significantly associated with mortality in those who did not know that they had the condition from which they later died (Jylha, 2009). The greater the information an individual has about their health the greater the predictive power of SRH (Jylha, 2009). In the present study, the participants knew if they had an inflammation-related disease, and this as well as the inflammation itself could have contributed to the association between SRH and mortality. It is very difficult to separate these two factors and this could have led to over-estimation of the contribution of inflammation per se to the association. The remaining association between SRH and mortality could also have been due in part to residual confounding, but the studies reviewed by Idler and Benyamini and others suggest that a relationship between SRH and mortality tends to persist even after these variables are allowed for (Idler and Benyamini, 1997). Similarly, though we could have used a wider range of biomarkers, as we have seen, some of the recent studies which have done this found that the aspect of health which is reflected in SRH went beyond that reflected in the biological measures chosen.

## Declaration of competing interest

None.
