# Supplementary material for: The role of inflammation in the relationship of self-rated health with mortality and implications for public health: Data from the English Longitudinal Study of Aging (ELSA)
Source: Brain Behav Immun Health. 2020 Sep 8;8:100139. doi: 10.1016/j.bbih.2020.100139 (PMC8474403; doi:10.1016/j.bbih.2020.100139)
Supplement: Multimedia component 1 [file mmc1.docx]

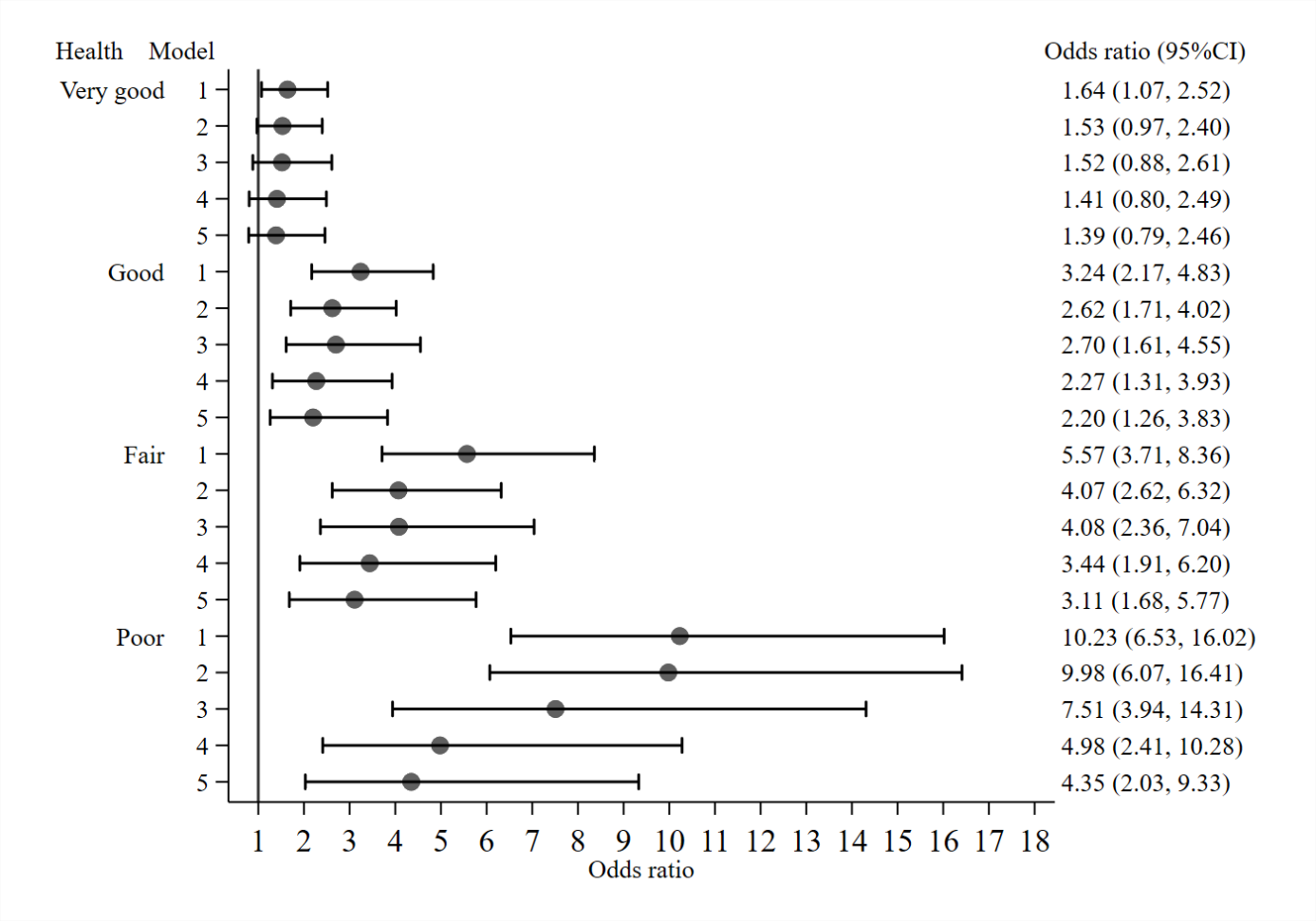


**Supplemental Figure 1**.  Odds ratio and 95% confidence interval for self-reported health from complete-case analysis stratified by models among men

Note. Men with excellent health were the reference groups. Model 1 is the unadjusted model. Model 2 controlled for age, ethnicity, socio-economic classification, total wealth quintiles, and marital status. Model 3 is the same as Model 2 with body mass index, smoking, alcohol consumption, positive social support, and physical activity being further controlled for. Model 4 is the same as Model 3 with C-reactive protein being further controlled for. Model 5 is the same as Model 4 with 7-CVD related diseases, other chronic diseases, cancer, and hypertension being further controlled for.


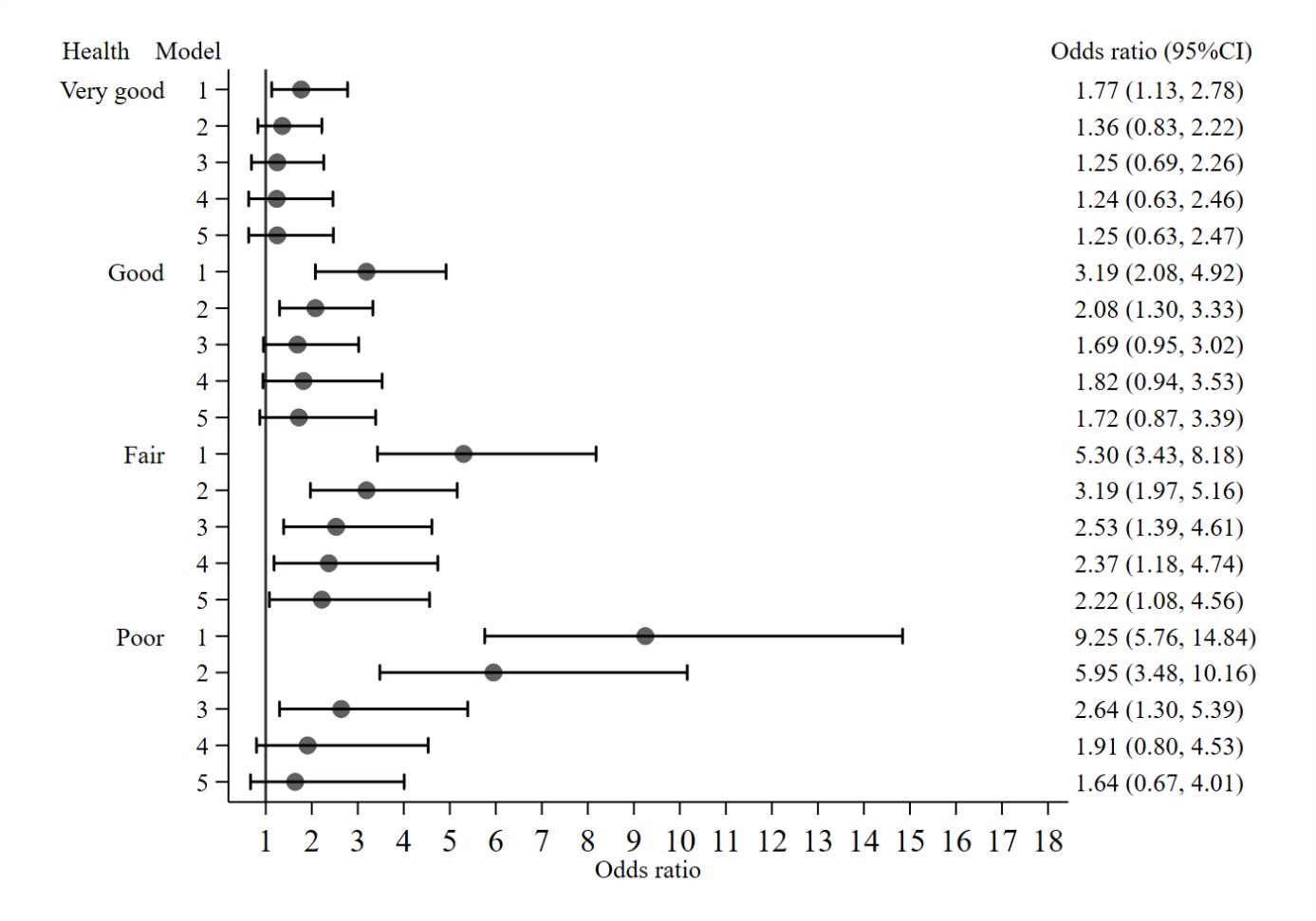


**Supplemental Figure 2**  Odds ratio and 95% confidence interval for self-reported health from complete-case stratified by models among women

Note. Women with excellent health were the reference groups. Model 1 is the unadjusted model. Model 2 controlled for age, ethnicity, socio-economic classification, total wealth quintiles, and marital status. Model 3 is the same as Model 2 with body mass index, smoking, alcohol consumption, positive social support, and physical activity being further controlled for. Model 4 is the same as Model 3 with C-reactive protein (CRP) being further controlled for. Model 5 is the same as Model 4 with 7-CVD related diseases, other chronic diseases, cancer, and hypertension being further controlled for.
